# Application of Digital Health Technologies in Cardiac Rehabilitation for Patients With Coronary Heart Disease: Scoping Review

**DOI:** 10.2196/85917

**Published:** 2026-04-29

**Authors:** Xinyu Zhu, Lei Liu, Yingjie Wang, Hongyuan Li, Min Zang, Jiayu Wang, Yu Tian, Zihan Li

**Affiliations:** 1 Liaoning University of Traditional Chinese Medicine, Shenyang, China; 2 School of Nursing, Liaoning University of Traditional Chinese Medicine, 79 Chongshan Road, Beita Street, Shenyang, China, 86 17824909908; 3 Department of Nursing, Affiliated Hospital of Liaoning University of Traditional Chinese Medicine, Shenyang, China; 4 Department of Nursing, Dalian Friendship Hospital, Dalian, China; 5 Liaoning Cancer Hospital & Institute, Shenyang, China

**Keywords:** digital health technologies, coronary heart disease, cardiac rehabilitation, scoping review, telemedicine

## Abstract

**Background:**

The high mortality and recurrence rates associated with coronary heart disease (CHD) impose substantial health care costs and economic burdens globally. Identifying effective interventions to improve patient outcomes is paramount. Digital health technologies (DHTs) offer novel solutions to overcome the challenge of low participation rates in traditional cardiac rehabilitation (CR).

**Objective:**

This review aims to systematically map the scope of application, intervention objectives, and evaluation metrics of DHTs in CR for patients with CHD, thereby providing a structured evidence base for future research and practice.

**Methods:**

This scoping review adheres to the Joanna Briggs Institute’s methodology and is reported according to the PRISMA-ScR (Preferred Reporting Items for Systematic Reviews and Meta-Analyses extension for Scoping Reviews) guidelines. A systematic search was conducted across 5 major databases, PubMed, Web of Science, Embase, Cochrane Library, and EBSCO, covering the period from inception to February 2026. Inclusion criteria were developed based on the participants, concept, and context framework. Studies focused on the application of various DHTs within CR settings for patients with CHD. Eligible literature comprised randomized controlled trials, quasi-randomized controlled trials, and longitudinal before-and-after studies published in peer-reviewed journals. Two researchers (XZ and ZL) independently conducted literature screening and data extraction. Findings were presented through a comprehensive narrative synthesis and evidence gap maps.

**Results:**

A total of 43 studies were included, predominantly randomized controlled trials (n=40). Findings revealed (1) diverse technological formats, categorized into 3 main types: digital health tools, real-time remote support, and asynchronous communication. Multitechnology combined interventions have become the mainstream model (36/43, 83.7%). (2) Intervention objectives were multifaceted, consolidating into 4 dimensions: motivation and guidance, knowledge and skills, monitoring and security, and social and group dynamics. (3) Evaluation metrics were multidimensional, encompassing clinical physiological indicators, health behaviors, patient-reported outcomes, service use rates, and technological feasibility. DHTs demonstrated positive effects in improving short-term physiological function and health behaviors; however, evidence remains insufficient regarding their impact on long-term clinical outcomes such as reducing adverse events.

**Conclusions:**

The innovation of this scoping review lies in integrating highly heterogeneous evidence to reveal the field’s evolution from isolated tools toward systematic, integrated solutions. Research confirms that DHTs effectively overcome temporal and spatial constraints, enhancing rehabilitation accessibility and engagement. They serve as crucial strategic tools for bridging geographical disparities in health care resources and advancing equity in cardiovascular health services. However, the evidence base remains limited, including insufficient long-term efficacy data and inadequate exploration of vulnerable populations such as older people and those with low digital literacy. Future research urgently requires large-scale, long-term follow-up clinical trials, alongside enhanced studies on adaptability for specific populations and considerations of health equity. This will propel digital CR toward greater scientific rigor, universal applicability, and precision.

## Introduction

### Background

The World Health Organization’s (WHO) report on the world’s top 10 causes of death indicates that cardiovascular disease (CVD) claims the highest number of lives, with coronary heart disease (CHD) accounting for 13% of global mortality [1]. Beyond the health burden, CVD, particularly CHD, imposes a significant economic strain. Globally, CHD accounts for 42% of total CVD expenditure, with annual per capita expenditure on CHD reaching 4.9% to 137.8% of per capita gross domestic product [2]. This underscores how CHD has become a “heavy burden” weighing upon individuals, families, society, and health care systems. More notably, despite substantial investment, the overall prognosis for patients with CHD has yet to improve effectively.

In response to this challenge, cardiac rehabilitation (CR) has demonstrated significant value as a comprehensive intervention. Research indicates that CR can reduce cardiovascular adverse events by 28%, 1-year readmission rates by 31%, and mortality by 24% while effectively reducing health care expenditure [3-5]. It is recommended as a class Ia evidence-based intervention in clinical guidelines [6,7]. However, participation rates in CR programs remain universally low among patients with CHD globally [8-10]. Rates range from 9.7% to 22.5% in Germany, 20% to 30% in the United States, and a mere 41.5% in the United Kingdom [8-10]. Asian nations present similarly unfavorable figures: Singapore at 12.3% and Japan and South Korea between 14% and 50% [4,11-13]. As the most populous developing nation, China has CR centers constituting merely 0.06% of all medical institutions, with underdeveloped regions accounting for a mere 8.8% [14]. Beyond awareness factors, limitations inherent to traditional CR, such as transport difficulties, time conflicts, and uneven resource distribution, constitute primary barriers to participation [15].

At this intersection of practical need and technological innovation, digital health technologies (DHTs) have transcended the constraints of conventional CR, forging novel pathways for its implementation [16]. In 2019, the WHO formally introduced the concept of DHTs, defining them as the field of developing and using digital technologies to disseminate health knowledge and facilitate related practices [17]. This encompasses applications of technologies such as the Internet of Things and artificial intelligence within health management [17]. Digital devices such as pedometers, accelerometers, and smartphones enable daily activity tracking, exercise intensity assessment, and personalized exercise guidance for patients with CHD [18,19]. Smart pillboxes and “digital pills” facilitate real-time monitoring of medication adherence [20,21]. Thus, DHTs overcome temporal and spatial constraints to deliver more accessible rehabilitation support. They effectively alleviate resource scarcity issues and hold promise for extending benefits to a broader population with CHD [22,23].

In summary, DHTs offer novel solutions to low CR participation rates. However, their highly heterogeneous delivery formats result in fragmented evidence. Compared to traditional review methodologies, scoping reviews can integrate heterogeneous evidence and define research boundaries [24-26]. Therefore, in this study, we use a scoping review approach to systematically collate evidence in this field, providing a holistic perspective for subsequent research and policy formulation. This aims to bridge gaps in cardiovascular health accessibility and advance the scientific, universal, and sustainable development of digital CR.

### Objectives and Research Questions

In this study, we aim to systematically review the scope of DHT applications in CR for patients with CHD through a scoping literature review methodology. It seeks to provide evidence-based guidance for the diversified development and effective implementation of future CR.

Our research will clarify (1) the application strategies and scenarios of existing digital technologies in CR for patients with CHD; (2) the key performance indicators determining the effectiveness of current DHT applications, alongside identifying their primary challenges; and (3) how DHTs can be more effectively applied to CR for patients with CHD and future research directions.

## Methods

### Overview

In this study, we strictly followed the Joanna Briggs Institute’s scoping review methodology framework to ensure methodological rigor and transparency in the research process [27,28]. This scoping review adhered strictly to a structured research process, using standardized methodologies to ensure the reliability of findings and their practical applicability. The research involved comprehensive systematic literature searches, data extraction, and evidence synthesis analysis, culminating in a narrative synthesis of studies concerning the application of DHTs in CR for patients with CHD. As a scoping review, our primary objective is to systematically map the current application, intervention formats, and outcome measures of DHTs in CR for patients with CHD. It does not evaluate intervention effectiveness or evidence quality grades; consequently, no rigorous methodological quality assessment of included studies was conducted [29]. Our review was reported according to the PRISMA-ScR (Preferred Reporting Items for Systematic Reviews and Meta-Analyses extension for Scoping Reviews) guidelines [26]. The PRISMA-ScR checklist is provided in Checklist 1.

### Eligibility Criteria

The inclusion criteria for this scoping review were based on the Joanna Briggs Institute Scope Review Methodology Guide and structured using the participants, concept, context framework [30]. The inclusion and exclusion criteria are presented in Textbox 1.

Textbox 1. **Inclusion and exclusion criteria. Eligibility criteria for the screening and inclusion process, including target population type, research setting, intervention measures, and study type.**

**Inclusion criteria**

- All patients diagnosed with coronary heart disease, irrespective of nationality, gender, or ethnic background. Coronary heart disease encompasses, but is not limited to, the following clinical presentations: stable angina pectoris, acute coronary syndromes (including unstable angina pectoris, non–ST-segment elevation myocardial infarction, and ST-segment elevation myocardial infarction), and patients who have undergone percutaneous coronary intervention or coronary artery bypass grafting.
- Focus on the various digital health technologies used in cardiac rehabilitation (CR), including but not limited to mobile health apps, wearable devices, telemedicine or remote monitoring platforms, educational modules delivered via web-based or online platforms, virtual reality, and text messaging.
- The context of interest pertains to the application of digital health technologies within CR, where such technologies serve as complementary, alternative, augmenting, or extended means to traditional CR. Their purpose is to support, optimize, or enhance the delivery of CR services. This context is not restricted to specific countries or health care systems, permitting the inclusion of studies from diverse cultural, geographical, or medical settings.
- The types of literature included are empirical studies, which must be published in peer-reviewed journals. The study designs encompass randomized controlled trials, quasi-randomized controlled trials, and longitudinal before-and-after studies.

**Exclusion criteria**

- Research participants who have undergone cardiac or cardiopulmonary transplantation or patients with chronic heart failure.
- Studies involving participants younger than 18 years of age.
- Non-English language literature, duplicated publications, gray literature, studies where the full text is unavailable, conference abstracts, review papers, or qualitative research.

### Information Sources

We conducted systematic searches of the following 5 electronic databases: PubMed, Web of Science (Clarivate), Embase (Elsevier), Cochrane Library (Wiley), and EBSCO (EBSCOhost). Searches were performed independently within each database interface, without using cross-database simultaneous search functionality.

### Search Strategy

The literature search process for this study was reported in accordance with the PRISMA-S (extension to the PRISMA Statement for Reporting Literature Searches in Systematic Reviews) guidelines [31]. The search strategy was independently developed by the research team based on the databases’ subject term lists, without direct adaptation or use of other published scoping review strategies. The complete search strategies for each database are detailed in Multimedia Appendix 1. The researchers first conducted a preliminary search in the PubMed database to expand the keywords and use MeSH to determine standardized subject terms. Following this, a search strategy was developed, and an initial search was conducted in PubMed, with a brief analysis of the results. Two researchers collaboratively developed the final search strategy, which was reviewed by a third researcher. The PubMed search strategy is detailed in Textbox 2. To maintain sensitivity, no restrictions were applied regarding study design, language, or publication type. The search time frame spanned from the inception of each database to February 2026. Furthermore, as this scoping review aimed to map published evidence to delineate the existing evidence base, clinical trial registries were excluded from the search.

Textbox 2. **PubMed search strategy.**

((((((((((((((((“Coronary Disease”[Mesh]) OR (“Myocardial Infarction”[Mesh])) OR (“Coronary Artery Disease”[Mesh])) OR (“Coronary Heart Disease*“[Title/Abstract])) OR (“Heart Attack*“[Title/Abstract])) OR (“Myocardial Infarct*“[Title/Abstract])) OR (“Cardiovascular Stroke*“[Title/Abstract])) OR (“acute coronary syndrome”[Title/Abstract])) OR (“angina pectoris”[Title/Abstract])) OR (“STEMI”[Title/Abstract])) OR (“NSTEMI”[Title/Abstract])) OR (“PCI”[Title/Abstract])) OR (“percutaneous coronary intervention”[Title/Abstract])) OR (“CABG”[Title/Abstract])) OR (“coronary artery bypass grafting”[Title/Abstract]))

AND

((((((((((((((((((((((((((((((((“Telemedicine”[Mesh]) OR (“Wearable Electronic Devices”[Mesh])) OR (“Digital Health”[Mesh])) OR (“Remote Patient Monitoring”[Mesh])) OR (“Text Messaging”[Mesh])) OR (“Virtual Medicine”[Title/Abstract])) OR (“Tele-Referral*“[Title/Abstract])) OR (“Mobile Health”[Title/Abstract])) OR (“mHealth”[Title/Abstract])) OR (“Telehealth”[Title/Abstract])) OR (“eHealth”[Title/Abstract])) OR (“Tele Intensive Care”[Title/Abstract])) OR (“Tele Care”[Title/Abstract])) OR (“Wearable Device*“[Title/Abstract])) OR (“Wearable Technolog*“[Title/Abstract])) OR (“Wearable Computer”[Title/Abstract])) OR (“Digital Health Technolog*“[Title/Abstract])) OR (“Health Technolog*“[Title/Abstract])) OR (“Short Message Service”[Title/Abstract])) OR (“digital therapeutics”[Title/Abstract])) OR (“smartwatch”[Title/Abstract])) OR (“fitness tracker”[Title/Abstract])) OR (“activity tracker”[Title/Abstract])) OR (“tele-rehabilitation”[Title/Abstract])) OR (“virtual care”[Title/Abstract])) OR (“mobile phone”[Title/Abstract])) OR (“cell phone”[Title/Abstract])) OR (“application”[Title/Abstract])) OR (“internet-based”[Title/Abstract])) OR (“web-based”[Title/Abstract])) OR (“online program”[Title/Abstract])))

AND

((((((((((((((((((“Cardiac Rehabilitation”[Mesh]) OR (“Secondary Prevention”[Mesh])) OR (“Cardiac Rehabilitation*“[Title/Abstract])) OR (“Cardiovascular Rehabilitation*“[Title/Abstract])) OR (“Secondary Prevention*“[Title/Abstract])) OR (“Disease Prevention*“[Title/Abstract])) OR (“Secondary Disease Prevention*“[Title/Abstract])) OR (“Early Therap*“[Title/Abstract])) OR (“Relapse Prevention*“[Title/Abstract])) OR (“exercise training”[Title/Abstract])) OR (“physical activity”[Title/Abstract])) OR (“lifestyle modification”[Title/Abstract])) OR (“behavior change”[Title/Abstract])) OR (“self-management”[Title/Abstract])) OR (“Exercise Therapy”[Title/Abstract])) OR (“Patient Education”[Title/Abstract])) OR (“Risk Factor Management”[Title/Abstract])) OR (“Medication Adherence”[Title/Abstract]))

### Selection of Sources

The literature retrieval and screening process were independently conducted by 2 researchers trained in evidence-based medicine and possessing cardiovascular research experience, with the entire procedure subject to third-party oversight. The specific workflow was as follows: (1) Search results from all databases were imported into EndNote (Clarivate) reference management software, which automatically identified and excluded duplicate records; (2) 2 researchers independently conducted an initial screening of the remaining literature based on predefined inclusion and exclusion criteria, reviewing titles and abstracts; (3) screening results were cross-checked; discrepancies were resolved through third-party discussion until consensus was reached; and (4) full-text evaluation of initially selected papers determined final inclusion in the study.

### Data Charting Process and Items

Data extraction was conducted using predesigned standardized forms by 2 researchers independently using Microsoft Excel to extract content covering the following core elements: (1) basic information: author, year of publication, and country; (2) study design: study type, sample size, follow-up duration, and control group configuration; (3) population characteristics: disease type; (4) intervention details: type of DHT, intervention duration, frequency of use, combination use, and combination method; (5) intervention objective; (6) outcome measures: primary outcomes and secondary outcomes; and (7) key findings: intervention effectiveness. In the event of disagreement during data extraction, a third researcher shall arbitrate the resolution.

## Results

### Selection of Sources of Evidence

**Figure 1.**
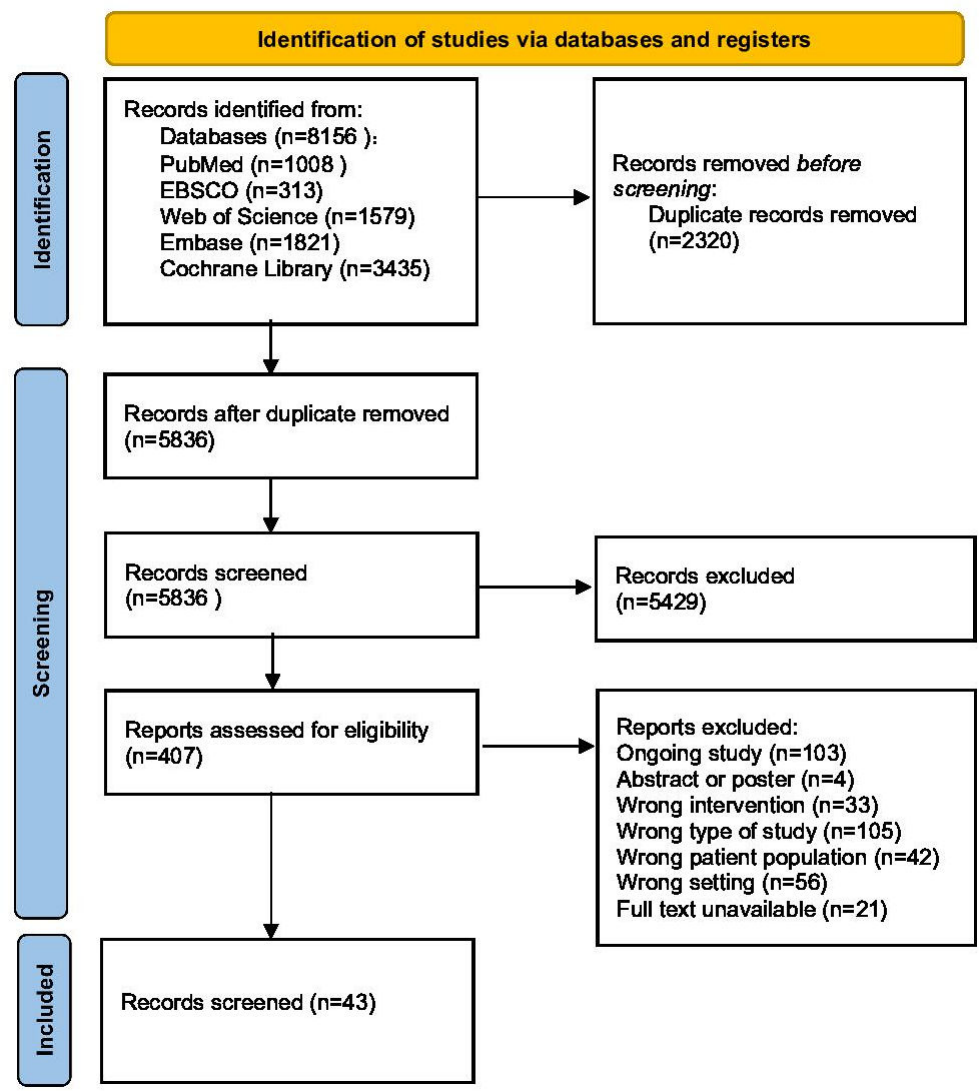
PRISMA flow diagram showing the identification of sources from databases and the screening and inclusion processes. PRISMA: Preferred Reporting Items for Systematic Reviews and Meta-Analyses.

As illustrated in Figure 1, this study strictly adhered to the PRISMA (Preferred Reporting Items for Systematic Reviews and Meta-Analyses) process for literature screening [32]. The initial search yielded 8156 publications. After removing 2320 duplicates using EndNote, 2 researchers (XZ and ZL) independently screened titles and abstracts. Based on predefined inclusion criteria, 5836 publications were excluded. The remaining 407 publications underwent full-text assessment, resulting in the exclusion of 364 publications that did not meet the requirements. Ultimately, 43 studies were included in the analysis.

### Characteristics of Sources of Evidence

**Figure 2.**
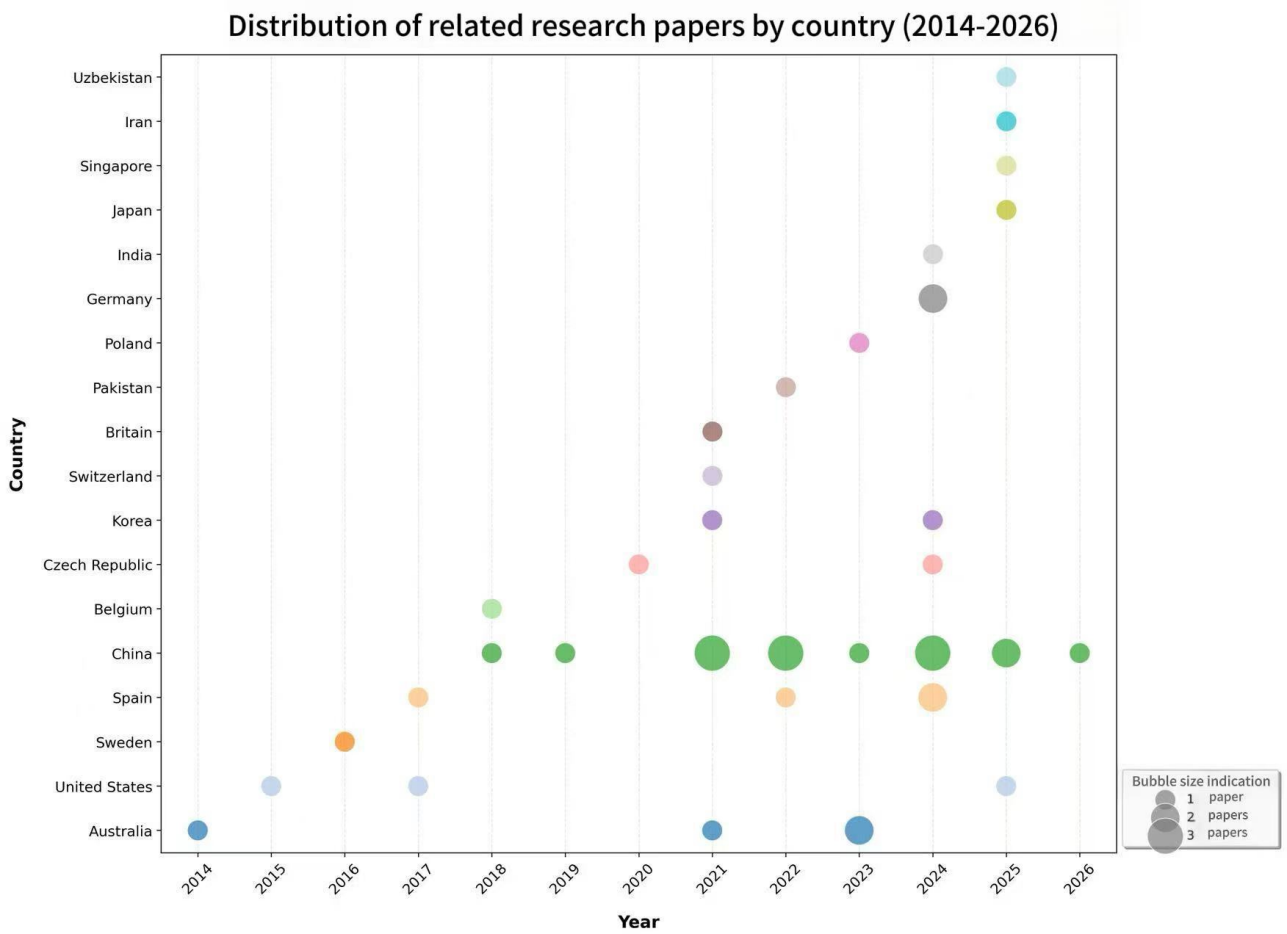
Publication trends for the 43 included studies by country and year (2014‐2026).

Figure 2 presents an overview of the 43 included studies by year and geographical distribution. The publication span of the included studies ranges from 2014 to 2026, with a marked increase in relevant research since 2021, with 60.5% of studies published from 2021 to 2026. The geographical coverage spans 18 countries, with China accounting for the highest proportion of studies at 34.9% (15/43) [19,33-46].

Of the 43 studies included in total, the vast majority used randomized controlled designs, comprising 33 randomized controlled trials [33,36,38,39,41,43-69] and 7 randomized controlled pilot studies [34,35,37,42,70-72]. Among the remaining studies, 2 were quasi-randomized controlled trials [40,73], and 1 was a controlled before-and-after study [74]. Detailed characteristics of the studies included in this scoping review are presented in Table 1.

**Table 1.** Study characteristics^a^.

Author (year)	Country	Design	Population (sample size)	Type and duration of intervention	Outcome measures	Statistically significant	Forms of intervention	Objectives of the intervention
Krzowski et al (2023) [47]	Poland	RCT^b^	Patients after acute myocardial infarction (n=100)	Intervention group: Standard rehabilitation+afterAMI app Control group: Standard rehabilitation Duration: 6 months	Primary: Rehospitalization or emergency department attendance within 6 months Secondary: Risk factor control, NT-proBNP^c^, disease knowledge	Partially valid	App	Health education Alerts and reminders Data monitor
Hong et al (2021) [33]	China	RCT	Patients with coronary artery disease (n=60)	Intervention group: Health IT system grounded in self-efficacy theory+self-monitoring devices+fortnightly telephone interviews Control group: Standard care (receiving identical intervention after a waiting period) Duration: 3-month intervention period, 6-month follow-up	Primary: Systolic blood pressure at 3 months Secondary: Self-management behaviors, quality of life, diastolic blood pressure	Valid	Website Wearable devices Telephone	Health education Data monitor Feedback Ensuring safety
Chan et al (2022) [34]	China	RCT pilot	Patients with stable coronary artery disease (n=139)	Intervention group: 15-minute face-to-face ZTEx introduction+ZTEX email+ZTEX app Control group: Equivalent duration and quantity of information on healthy eating and breathing exercises Duration: 12 weeks	Primary: Physical activity (IPAQ^d^), physical fitness Secondary: Self-efficacy in exercise, well-being, quality of life	Valid	App Social media platform	Health education Data monitor Alerts and reminders
Varnfield et al (2014) [48]	Australia	RCT	Patients after myocardial infarction (n=120)	Intervention group: CAP-CR Control group: Traditional centralized CR^e^ Duration: 6-week intervention period, followed by a 6-month maintenance phase	Primary: Rehabilitation acceptance rate, adherence rate, completion rate Secondary: Lifestyle factors, clinical indicators, quality of life	Valid	App Wearable devices Digital video	Health education Data monitor Personalization Goal setting
Duan et al (2018) [35]	China	RCT pilot	Patients with coronary artery disease (n=136)	Intervention group: An 8-week web-based intervention grounded in the health action process approach (HAPA) model Control group: A waiting-list control group Duration: 8 weeks	Primary: Physical activity, fruit and vegetable intake Secondary: Healthy lifestyle, social cognitive indicators, health outcomes	Valid	Website	Health education Data monitor Goal setting Peer effect Feedback
Xu et al (2024) [36]	China	RCT	Patients with CHD^f^ after PCI^g^ (n=147)	Intervention group: Remote rehabilitation strategy based on the SCeiP^h^ model Control group: Standard exercise rehabilitation Duration: 3 months	Primary: Exercise adherence (number of days, duration) Secondary: CR awareness, exercise program, exercise commitment	Valid	Social media platform Wearable devices Telephone	Health education Data monitor Feedback Remotely adjust prescription
Avila et al (2018) [49]	Belgium	RCT	Patients with coronary artery disease (n=90)	Intervention group 1: Home-based rehabilitation+remote monitoring Intervention group 2: In-hospital rehabilitation Control group: Standard care Duration: 12 weeks	Primary: Peak oxygen uptake (VO_2_ max) Secondary: Physical activity, risk factors, quality of life	Valid	Website Wearable devices Email Telephone	Health education Data monitor Feedback Personalization Goal setting
Nishio et al (2025) [70]	Japan	RCT pilot	Patients with coronary artery disease (n=50)	Intervention group: Wearable device + real-time monitoring+weekly text message and monthly videoconference guidance Control group: Wearable device only Duration: 12 weeks	Primary: Changes in VO_2_ max and anaerobic threshold oxygen uptake Secondary: Daily activities, anxiety, quality of life	Partially valid	Wearable devices SMS text messaging Remote counseling sessions	Health education Data monitor Personalization
Li et al (2022) [37]	China	RCT pilot	Patients with coronary artery disease (n=300)	Intervention group: Traditional follow-up+self-management app Control group: Traditional hospital follow-up Duration: 1 year	Primary: Proportion of patients receiving all guideline-recommended medications at 12 months Secondary: Proportion receiving medication at 6 months, lipid control rate, blood pressure control rate	Valid	App	Health education Data monitor Personalization Alerts and reminders
Cruz-Cobo et al (2024) [50]	Spain	RCT	Patients with acute coronary syndrome after PCI (n=300)	Intervention group: Standard care + eMOTIVA app Control group: Standard care Duration: 6 months	Primary: Mediterranean diet adherence, physical activity, sedentary time, functional capacity, smoking cessation, disease knowledge, app satisfaction Secondary: No	Valid	App Remote counseling sessions Digital video SMS text messaging	Health education Data monitor Personalization Alerts and reminders Goal setting Feedback Gamification Reward mechanism
Bernal-Jiménez et al (2024) [51]	Spain	RCT	Patients with CHD after PCI (n=128)	Intervention group: Standard care+Interactive mHealth App (EVITE app) Control group: Standard care Duration: 9 months	Primary: Mediterranean diet adherence, food frequency, physical activity, smoking, knowledge, treatment adherence, quality of life, satisfaction Secondary: No	Valid	App Remote counseling sessions Email Telephone SMS text messaging	Health education Data monitor Personalization Alerts and reminders Goal setting Feedback
Bae et al (2021) [52]	Korea	RCT	Patients after the first PCI (n=879)	Intervention group: Standard care + website + 4 weekly lifestyle text messages Control group: Standard care Duration: 6 months	Primary: Low-density lipoprotein cholesterol, systolic blood pressure, BMI Secondary: Lifestyle modifications, adherence to health behaviors	Invalid	Wearable devices SMS text messaging	Health education Alerts and reminders
Su and Yu (2021) [38]	China	RCT	Patients with coronary artery disease (n=146)	Intervention group: Standard care+NeCR Control group: Standard care Duration: 12 weeks	Primary: Lifestyle behavior changes Secondary: Self-efficacy, quality of life, physiological indicators	Valid	Social media platform Remote counseling sessions Digital video	Health education Alerts and reminders Feedback Goal setting Social support Data monitor Emotional support or counseling
Dodson et al (2025) [53]	United States	RCT	Patients aged 65 years or older with ischemic heart disease (n=400)	Intervention group: mHealth-CR software on tablet devices+remote monitoring+weekly telephone guidance Control group: standard care Duration: 3 months	Primary: Change in 6MWD^i^ from baseline to 3 months Secondary: Health status, residual angina, impairment in activities of daily living	Partially valid	App Remote counseling sessions Telephone Wearable devices	Health education Feedback Goal setting Data monitor
Dorje et al (2019) [19]	China	RCT	Patients with CHD after PCI (n=312)	Intervention group: SMART-CR/SP Control group: Standard care Duration: 6 months	Primary: Change in 6MWD at 2 months and 6 months Secondary: No	Valid	Social media platform Wearable devices Telephone Remote counseling sessions	Health education Feedback Data monitor Alerts and reminders
Hisam et al (2022) [54]	Pakistan	RCT	After an acute coronary syndrome (n=160)	Intervention group: Standard care+MCard Control group: Standard care Duration: 24 weeks	Primary: Health-related quality of life Secondary: No	Valid	App Wearable devices SMS text messaging	Health education Data monitor Alerts and reminders Emotional support or counseling
Zheng et al (2024) [39]	China	RCT	Patients with CHD after PCI (n=106)	Intervention group: Standard care+HCT Control group: Standard care Duration: 3 months	Primary: 6-minute walk test, quality of life (SF-12^j^), disease burden (FBIS^k^), cardiac function (LVEF^l^) Secondary: No	Valid	App Wearable devices Telephone	Health education Data monitor Goal setting Remotely adjust prescription Feedback
Ma et al (2021) [40]	China	Q-RCT^m^	Patients with CHD after PCI (n=335)	Intervention group: HBCR Control group: Routine care Duration: 42 months	Primary: Incidence of major adverse cardiovascular and stroke events Secondary: Exercise capacity, quality of life, risk factor control	Valid	App Wearable devices Digital video	Health education Data monitor Training courses Ensuring safety Alerts and reminders
Xu et al (2024) [41]	China	RCT	Patients with coronary artery disease (n=108)	Intervention group 1 (individual): Gamified intervention Intervention group 2 (team): Gamification+social interaction Control group: Daily step target Duration: 12-week intervention, 12-week follow-up	Primary: Daily step count variation, proportion of days achieving target Secondary: Autonomy, sense of connection, intrinsic motivation	Valid	App	Goal setting Feedback Social support Reward mechanism Gamification
Widmer et al (2017) [55]	United States	RCT	Patients with acute coronary syndrome after PCI (n=80)	Intervention group: Standard CR+DHI Control group: Standard CR Duration: 3-month intervention, 6-month outcome assessment	Primary: Related emergency department visits and readmissions Secondary: Risk factors and lifestyle factors at 90 days	Partially valid	Website Remote counseling sessions	Health education Data monitor
Yudi et al (2021) [56]	Australia	RCT	Patients with acute coronary syndromes (n=206)	Intervention group: Standard care+S-CRP Control group: Standard care Duration: 8 weeks	Primary: Change in 6MWD at 8 weeks Secondary: CR participation, risk factors, psychological indicators	Valid	App Remote counseling sessions	Health education Data monitor Feedback Ensuring safety Personalization
Gallagher et al (2023) [57]	Australia	RCT	Patients with coronary artery disease (n=390)	Intervention group: MyHeartMate app Control group: Standard care Duration: 6 months	Primary: Self-reported physical activity (METs^n^) Secondary: Blood lipids, blood pressure, BMI, smoking	Invalid	App Digital video Email	Gamification Data monitor Social support Reward mechanism Training courses
Wohlfahrt et al (2024) [71]	Czech Republic	RCT pilot	Patients after myocardial infarction (n=64)	Intervention group: Smartwatch step tracking+remote monitoring by nurses Control group: 150 minutes per week of moderate-intensity exercise recommendation Duration: 3 months, followed by crossover after 3 months	Primary: VO_2_ max Secondary: Body weight, 6MWD, quality of life	Valid	Wearable devices Telephone	Data monitor Alerts and reminders Real-time monitoring Goal setting
Ramachandran et al (2025) [72]	Singapore	RCT pilot	Patients after acute myocardial infarction (n=50)	Intervention group: Home-based remote rehabilitation Control group: Centralized CR Duration: 6 weeks	Primary: Use of CR Secondary: Functional capacity, risk factors, self-reported behaviors	Valid	App Website Wearable devices Telephone Remote counseling sessions	Health education Training courses Data monitor Alerts and reminders Goal setting Remotely adjust prescription
Jo et al (2024) [58]	Korea	RCT	Patients after acute myocardial infarction (n=48)	Intervention group: Mobile app-based rehabilitation Control group: Conventional home-based rehabilitation+biweekly telephone supervision Duration: 6 weeks	Primary: VO_2_ max Secondary: Resting heart rate, blood pressure, quality of life, psychological indicators	Invalid	App Telephone Wearable devices	Health education Data monitor Goal setting Ensuring safety Real-time monitoring Training courses
Fallah et al (2025) [59]	Iran	RCT	Patients with myocardial infarction (n=144)	Intervention group: HAPA-based mobile app Control group: No specific intervention Duration: 8 weeks	Primary: Physical activity (IPAQ) Secondary: HAPA-related psychological constructs	Valid	App	Health education Training courses Feedback Social support Personalization
Li et al (2023) [42]	China	RCT pilot	Patients with acute myocardial infarction after PCI (n=60)	Intervention group: 5G IoT platform Control group: Conventional CR training within the hospital. Duration: 3 months.	Primary: Cardiorespiratory fitness (VO_2_ max, MET) Secondary: Physiological indicators, psychological indicators, adherence, satisfaction.	Valid	Website Wearable devices	Health education Training courses Feedback Remotely adjust prescription Personalization Emotional support or counseling
Waranski et al (2024) [73]	Germany	Q-RCT	Patients with coronary artery disease (n=169)	Intervention group: Personalized messages (twice weekly) Control group: Routine outpatient care Duration: 6 months	Primary: Routine physical activity (≥150 minutes per week) and daily activities at 6 months Secondary: Psychological indicators, self-efficacy, quality of life	Valid	App SMS text messaging Telephone	Health education Alerts and reminders Goal setting Personalization
Ni et al (2022) [43]	China	RCT	Patients with coronary artery disease (n=230)	Intervention group: WeChat+Message Express Control group: WeChat only Duration: 60-day intervention, 30-day follow-up	Primary: Medication Adherence Score (Voils Extent Scale) Secondary: Heart rate, systolic blood pressure, diastolic blood pressure	Valid	SMS text messaging Social media platform	Health education Alerts and reminders
Liu et al (2026) [44]	China	RCT	Patients after PCI (n=180)	Intervention group: Standard rehabilitation+eHealth platform based on the Persuasive systems design (PSD) model Control group: Standard rehabilitation Duration: 12-week intervention, 12-week follow-up	Primary: Physical activity level (IPAQ) Secondary: Exercise endurance (6MWD), self-perceived fatigue, exercise self-efficacy, quality of life	Valid	App Social media platform Telephone	Health education Alerts and reminders Training courses
Bruggmann et al (2021) [60]	Switzerland	RCT	Patients with acute coronary syndromes (n=60)	Intervention group: Routine care+viewing of interactive video+brief interview with pharmacist Control group: Routine care Duration: 6 months	Primary: Differences in medication adherence at 1, 3, and 6 months (ARMS^o^) Secondary: Disease knowledge, readmission, emergency department visits, satisfaction	Partially valid	Website	Health education Training courses Feedback
Zhang et al (2025) [45]	China	RCT	Patients with coronary artery disease (n=62)	Intervention group: Smartwatch-assisted CR Control group: Standard CR Duration: 3 months	Primary: HBCR adherence (HETAQ^p^) Secondary: cardiopulmonary function, anxiety, depression, quality of life	Valid	Wearable devices App	Health education Real-time monitoring Data monitor Feedback Alerts and reminders
Patterson et al (2023) [61]	Australia	RCT	Patients with coronary artery disease (n=120)	Intervention group: Conventional rehabilitation+Vire app Control group: Conventional rehabilitation Duration: 12 months	Primary: Nonelective hospital admissions and emergency department visits Secondary: Sedentary behavior, BMI, waist circumference, quality of life, cost-effectiveness	Invalid	Wearable devices App	Data monitor Feedback Alerts and reminders Personalization
Batalik et al (2020) [62]	Czech Republic	RCT	Patients with coronary artery disease (n=56)	Intervention group: Home-based remote rehabilitation Control group: Routine outpatient rehabilitation Duration: 12 weeks	Primary: VO_2_ max Secondary: Quality of life (SF-36^q^), training adherence	Invalid	Wearable devices App Telephone	Data monitor Feedback Emotional support or counseling
Dalli Peydró et al (2022) [63]	Spain	RCT	Patients with acute coronary syndromes (n=67)	Intervention group: Remote CR Control group: Center-based CR Duration: 8 weeks	Primary: Self-reported physical activity (IPAQ) Secondary: VO_2_ max, blood lipids, body weight, quality of life, time to return to work	Valid	Wearable devices App	Data monitor Feedback Personalization Training courses
Bravo-Escobar et al (2017) [64]	Spain	RCT	Patients with stable, intermediate-risk coronary artery disease (n=28)	Intervention group: Weekly hospital sessions+3 home training sessions Control group: Routine hospital rehabilitation (3 sessions per week) Duration: 2 months	Primary: Physical fitness, risk profile, cardiovascular complications, quality of life Secondary: No	Partially valid	Wearable devices App	Data monitor Feedback Goal setting
Widmer et al (2015) [74]	United States	CBA^r^	Patients after myocardial infarction (n=42)	Intervention group 1: During CR+PHA Intervention group 2: Following CR+PHA Control group: Standard CR during the corresponding time period Duration: 3 months	Primary: Changes in risk factors and readmissions or emergency department visits after 3 months Secondary: No	Valid	Website	Data monitor Feedback Alerts and reminders Health education
Johnston et al (2016) [65]	Sweden	RCT	Patients after myocardial infarction (n=174)	Intervention group: Full-featured CR app Control group: Simplified version app Duration: 6 months	Primary: Nonadherence score based on app records Secondary: Risk factors, quality of life, device satisfaction	Valid	App SMS text messaging Telephone	Data monitor Feedback Alerts and reminders Health education
Kumar et al (2024) [66]	India	RCT	Patients after CABG (n=40)	Intervention group: eMedia-supported exercise rehabilitation Control group: Standard care Duration: 12 weeks	Primary: Functional capacity (6MWT), quality of life (WHOQOL-BREF^s^), physical activity (GPAQ^t^) Secondary: No	Valid	Website App	Feedback Health education Ensuring safety Data monitor
Bretschneider et al (2024) [67]	Germany	RCT	Patients with coronary artery disease (n=354)	Intervention group: Standard care plus Mebix Control group: Standard care Duration: 12 months	Primary: Disease-specific quality of life (HeartQoL^u^) and body weight Secondary: Cardiovascular risk, occupational prognosis	Valid	App SMS text messaging Telephone	Data monitor Feedback Alerts and reminders Health education Training courses
Herring et al (2021) [68]	Britain	RCT	Patients with coronary artery disease (n=291)	Intervention group: 2 structured educational sessions+follow-up text message support Control group: Standard care Duration: 12 months	Primary: Changes in overall physical activity at 12 months (GENEActiv) Secondary: Functional, cardiovascular, biochemical, and patient-reported outcomes	Invalid	Group meeting SMS text messaging	Health education Alerts and reminders
Li et al (2025) [46]	China	RCT	Patients with coronary artery disease (n=294)	Intervention group: Personalized face-to-face education+i-CARE app+pedometer Control group: Standard care+pedometer Duration: 6 months	Primary: Self-care behavior in CHD (SC-CHDI^v^) Secondary: Health status, quality of life, physiological indicators	Valid	App Wearable devices	Data monitor Feedback Health education Real-time monitoring Social support Personalization
Khikmatova Madina et al [69] (2025)	Uzbekistan	RCT	Patients after myocardial infarction (n=300)	Intervention group: Standard care+wearable activity monitoring device and accompanying app Control group: Standard care Duration: 6 months	Primary: Rehabilitation adherence Secondary: Readmission rate, mortality rate, ejection fraction, exercise capacity	Valid	App Wearable devices Remote counseling sessions	Health education Data monitor Real-time monitoring Alerts and reminders

a Information regarding authors, publication year, participants, study design, study outcomes, and methodology for 43 studies. Digital health technology intervention name: zero-time exercise (ZTEx), care assessment platform of cardiac rehabilitation (CAP-CR), nurse-led eHealth cardiac rehabilitation (NeCR), mobile health cardiac rehabilitation (mHealth-CR), smartphone and social media–based cardiac rehabilitation and secondary prevention (SMART-CR/SP), mobile health augmented cardiac rehabilitation (MCard), home-based cardiac telerehabilitation (HCT), home-based cardiac rehabilitation (HBCR), digital health intervention (DHI), smartphone-based cardiac rehabilitation program (S-CRP), fifth generation mobile communication technology Internet of Things platform (5G IoT platform), persuasive systems design (PSD), coronary artery bypass grafting (CABG), internet-based cardiac rehabilitation enhancement (i-CARE).

b RCT: randomized controlled trial.

c NT-proBNP: N-terminal pro-brain natriuretic peptide.

d IPAQ: International Physical Activity Questionnaire.

e CR: cardiac rehabilitation.

f CHD: coronary heart disease.

g PCI: percutaneous coronary intervention.

h SCeiP: Self-Monitoring, Coaching, e-Health, Interactive Feedback, and Personalization.

i 6MWD: 6-minute walk distance.

j SF-12: Short Form 12 Health Survey.

k FBIS: Framingham Burden of Illness Scale.

l LVEF: left ventricular ejection fraction.

m Q-RCT: quasi-randomized controlled trial.

n MET: metabolic equivalents of task.

o ARMS: Adherence to Refills and Medications Scale.

p HETAQ: Home-Based Cardiac Rehabilitation Adherence Questionnaire.

q SF-36: Short Form 36 Health Survey.

r CBA: controlled before-after study.

s WHOQOL-BREF: World Health Organization Quality of Life-BREF.

t GPAQ: Global Physical Activity Questionnaire.

u HeartQoL: Heart Disease-Specific Quality of Life Questionnaire.

v SC-CHDI: Self-Care Behaviour in Coronary Heart Disease Inventory.

### Results of Individual Sources of Evidence

In this study, we analyzed data from 43 research papers and generated an evidence gap map (Table 2) illustrating the application forms and objectives of DHT interventions.

**Table 2.** Evidence gap analysis of digital health technologies in cardiac rehabilitation for patients with coronary heart disease, based on 43 included studies.

Author (year)	Forms of intervention										Objectives of the intervention															
	Wearable devices	Application	Website	Digital video	Social media platform	Telephone	Group meeting	Remote counseling sessions	Email	SMS text messaging	Goal setting	Feedback	Reward mechanism	Gamification	Alerts and reminders	Personalization	Health education	Training courses	Data monitor	Real-time monitoring	Ensuring safety	Remotely adjust prescription	Peer effect	Social support	Emotional support	Counseling
Krzowski et al (2023) [47]		✓													✓		✓		✓							
Hong et al (2021) [33]	✓		✓			✓						✓					✓		✓		✓					
Chan et al (2022) [34]		✓			✓										✓		✓		✓							
Varnfield et al (2014) [48]	✓	✓		✓							✓					✓	✓		✓							
Duan et al (2018) [35]			✓								✓	✓					✓		✓				✓			
Xu et al (2024) [36]	✓				✓	✓						✓	✓	✓			✓		✓			✓		✓		
Avila et al (2018) [49]	✓		✓			✓			✓		✓	✓				✓	✓		✓							
Nishio et al (2025) [70]	✓							✓		✓						✓	✓		✓							
Li et al (2022) [37]		✓													✓	✓	✓		✓							
Cruz-Cobo et al (2024) [50]		✓		✓				✓		✓	✓	✓	✓	✓	✓	✓	✓		✓							
Bernal-Jiménez et al (2024) [51]		✓				✓		✓	✓	✓	✓	✓			✓	✓	✓		✓							
Bae et al (2021) [52]	✓									✓					✓		✓									
Su and Yu (2021) [38]				✓	✓			✓			✓	✓			✓		✓		✓					✓	✓	✓
Dodson et al (2025) [53]	✓	✓				✓		✓			✓	✓					✓		✓							
Dorje et al (2019) [19]	✓				✓	✓		✓				✓			✓		✓		✓							
Hisam et al (2022) [54]	✓	✓								✓					✓		✓		✓						✓	✓
Zheng et al (2024) [39]	✓	✓				✓					✓	✓					✓		✓			✓				
Ma et al (2021) [40]	✓	✓		✓											✓		✓	✓	✓		✓					
Xu et al (2024) [41]		✓									✓	✓														
Widmer et al (2017) [55]			✓					✓									✓		✓							
Yudi et al (2021) [56]		✓						✓				✓				✓	✓		✓		✓					
Gallagher et al (2023) [57]		✓		✓					✓				✓	✓				✓	✓					✓		
Wohlfahrt et al (2024) [71]	✓					✓					✓				✓				✓	✓						
Ramachandran et al (2025) [72]	✓	✓	✓			✓		✓			✓				✓		✓	✓	✓			✓				
Jo et al (2024) [58]	✓	✓				✓					✓						✓	✓	✓	✓	✓					
Fallah et al (2025) [59]		✓										✓				✓	✓	✓						✓		
Li et al (2023) [42]	✓		✓									✓				✓	✓	✓				✓			✓	✓
Waranski et al (2024) [73]		✓				✓				✓	✓				✓	✓	✓									
Ni et al (2022) [43]					✓					✓					✓		✓									
Liu et al (2026) [44]		✓			✓	✓									✓		✓	✓								
Bruggmann et al (2021) [60]			✓									✓					✓	✓								
Zhang et al (2025) [45]	✓	✓										✓			✓		✓		✓	✓						
Patterson et al (2023) [61]	✓	✓										✓			✓	✓			✓							
Batalik et al (2020) [62]	✓	✓				✓						✓							✓						✓	✓
Dalli Peydró et al (2022) [63]	✓	✓										✓				✓		✓	✓							
Bravo-Escobar et al (2017) [64]	✓	✓									✓	✓							✓							
Widmer et al (2015) [74]			✓									✓			✓		✓		✓							
Johnston et al (2016) [65]		✓				✓				✓		✓			✓		✓		✓							
Kumar et al (2024) [66]		✓	✓									✓					✓		✓		✓					
Bretschneider et al (2024) [67]		✓				✓				✓		✓			✓		✓	✓	✓							
Herring et al (2021) [68]							✓			✓					✓		✓									
Li et al (2025) [46]	✓	✓										✓				✓	✓		✓	✓				✓		
Khikmatova et al (2025) [69]	✓	✓						✓							✓		✓		✓	✓						

### The Form of DHT in CR for Patients With CHD

The 43 studies included in this research demonstrate that DHTs exhibit significant diversity in their application within CR for patients with CHD. These technologies can be categorized into 3 core groups (Table 3): digital health tools, real-time remote support, and asynchronous communication. Among these, digital health tools represent the most prevalent intervention form, enabling patients with CHD to undertake self-management and monitoring primarily through devices or software. This includes apps (28/43, 65.1%) [34,37,39-41,44-48,50,51,53,54,56-59,61-67,69,72,73], wearable devices (22/43, 51.1%) [19,33,36,39,40,42,45,46,49,52-54,58,61-64,69-72], websites (9/43, 20.9%) [33,35,42,49,55,60,66,72,74], and social media platforms (6/43, 13.9%) [19,34,36,38,43,44]. Wearable devices encompass smartwatches, heart rate monitors, fitness trackers, and pedometers, primarily used for real-time monitoring of physiological indicators and exercise data. Real-time remote support involves direct interpersonal interaction via voice or video, covering telephone (15/43, 34.8%) [19,33,36,38,39,44,49,51,53,57,62,65,67,72,73], remote counseling sessions, and group meetings. Asynchronous communication delivers reminders, education, and support through non–real-time information exchange, chiefly via SMS text messaging (10/43, 23.2%) [43,50-52,65,67,68,70,73] and email. As shown in Table 2, multitechnology combined interventions have become the predominant model. A substantial 83.7% (36/43) of studies used combinations of 2 or more digital technologies, such as “app+wearable device” [39,40,45,46,48,53,54,58,61-64,69,72] and “social media platform+wearable device+telephone” [19,36]. Some studies further integrated digital technologies with traditional rehabilitation methods like face-to-face guidance and offline education, forming blended online-offline rehabilitation models. This landscape not only reflects varying levels of technological support, from standalone tools to interpersonal interactions, but also signals the trend toward systematized and diversified digital CR.

**Table 3.** A total of 3 categories and 10 specific forms of digital health technology application in cardiac rehabilitation interventions.

Type	Core functionality	Content
Digital health tools	Provide patients with tools for independent health management through a technology platform, emphasizing self-monitoring and personalized interaction.	Wearable devices App Website Digital video Social media platform
Real-time remote support	Provides real-time, person-to-person professional support or peer interaction through synchronous communication technology, with high interactivity.	Telephone Group meeting Remote counseling sessions
Asynchronous communication	Reminders, education, and support are provided through non–real-time information transmission methods, which are flexible and not restricted by time or space.	Email SMS text messaging

### The Objectives of DHT in CR for Patients With CHD

From the perspective of intervention objectives, the application of DHTs in CHD rehabilitation exhibits distinct functional stratification. Health education (36/43, 83.7%) [19,33-40,58-60,65-70,72-74], data monitor (34/43, 79.1%) [19,33-40,45-51,53-58,61-67,69-74], and reminders and alerts (18/43, 41.9%) [19,34,37,38,40,43-45,47,50-52,54,55,71-73] form the core functional layer, each accounting for over 80% of applications in the included studies. Feedback, goal setting, and personalized interventions constitute the secondary core functional layer, with application rates ranging between 50% and 70%. Additionally, some studies integrated distinctive features such as gamification, reward mechanisms, social support, emotional support or counseling, and remotely adjust prescription to address patients’ diverse rehabilitation needs. Building upon this, this study systematically categorized the intervention objectives across 43 publications, identifying 15 specific types grouped into 4 major categories: first, motivation and guidance, encompassing goal setting, feedback, reward mechanisms, gamification, and reminders and alerts, aimed at incentivizing patients with CHD to complete rehabilitation behaviors and enhance adherence; second, foundation of knowledge and skills, centered on health education and training courses to help patients with CHD build the knowledge base and self-management capabilities required for disease management; third, monitoring and security, including data monitoring, real-time monitoring, and ensuring safety for physiological indicator tracking, risk assessment, and safety protection during rehabilitation; and fourth, social and group dynamics, leveraging peer effects and social support mechanisms to use social relationships and group interactions to promote patient adherence to rehabilitation behaviors. The specific composition is detailed in Table 4. As shown in Table 2, health education emerged most frequently, underscoring the central role of knowledge transfer in contemporary digital rehabilitation practice. Notably, the vast majority of studies adopted multipurpose integrated intervention strategies, organically combining educational, motivational, monitoring, and social support functions rather than relying on single technological approaches. It is precisely this composite application model that has transformed DHTs from fragmented tools into systematic rehabilitation support systems, significantly enhancing the holistic nature and continuity of rehabilitation interventions.

**Table 4.** A total of 4 key intervention objectives and 15 specific types of digital health technologies in cardiac rehabilitation for individuals with coronary heart disease.

Type	Core objective	Content
Motivation and guidance	Motivate patients and guide them to complete specific behaviors.	Goal setting Feedback Reward mechanism Gamification Alerts and reminders Personalization
Foundation of knowledge and skills	Provide necessary information and cultivate patients’ self-management skills.	Health education Training courses
Monitoring and security	Track data, assess risks, and provide a safety net.	Data monitor Real-time monitoring Ensuring safety Remotely adjust prescription
Social and group dynamics	Using social relationships and group dynamics to promote patient adherence and change.	Peer effect Social support Emotional support or counseling

### Evaluation Criteria of DHT in CR for Patients With CHD

The outcome measures included in the study encompass 5 major categories: clinical physiological indicators, rehabilitation behavioral indicators, patient-reported outcomes, rehabilitation service use rates, and technical feasibility. Clinical physiological indicators include peak oxygen uptake, 6-minute walk distance, and blood pressure. Rehabilitation behavioral indicators include exercise adherence, medication adherence, physical activity levels, dietary adherence, and sedentary time. Patient-reported outcomes encompassed quality of life, self-efficacy, anxiety and depression levels, disease knowledge, and rehabilitation satisfaction. Rehabilitation service use metrics included rehabilitation acceptance rate, adherence rate, completion rate, readmission rate, and emergency department visit rate. Technical feasibility referred to patient satisfaction with the DHT used.

### Clinical Efficacy and Physiological Indicators

Clinical efficacy and physiological indicators constitute the core dimensions for evaluating the effectiveness of DHTs, with over 60% of studies incorporating them as primary outcomes. These encompass 3 specific levels: cardiopulmonary function, physical capacity and strength, and clinical end-point events. Cardiopulmonary function stands as the most critical indicator, with peak oxygen uptake [39,42,44,49,58,66,70,71] and 6-minute walk distance [42,49,58,62,63,70,71] being the most widely applied measures. Combining remote monitoring, wearable devices, and online guidance can effectively improve patients’ cardiopulmonary function, as measured by peak oxygen uptake and 6-minute walk distance [49,70]. Physical function and strength serve as supplementary dimensions, encompassing muscle endurance and overall physical capacity. Dalli Peydró et al [63] confirmed that remote rehabilitation improves patients’ physical activity capabilities. Regarding clinical end points, over 20 studies evaluated blood pressure, lipid profiles, N-terminal pro-brain natriuretic peptide, and left ventricular ejection fraction. Li et al [37] found that app-based interventions increased lipid control rates; however, no consistent conclusions have emerged regarding long-term outcomes such as readmission rates and major adverse cardiovascular events. For instance, Krzowski et al [47] did not demonstrate a significant advantage of digital interventions in reducing readmission rates, suggesting that further research is needed to substantiate long-term efficacy.

### Health Behavior and Lifestyle

Health behaviors and lifestyle constitute core factors in improving the long-term prognosis of patients with CHD, with over half of the studies incorporating them into evaluations. These are categorized into 2 dimensions: exercise behavior and daily lifestyle. Regarding exercise behavior, key indicators include physical activity levels, exercise adherence, number of exercise days, and duration. Xu et al [41] demonstrated in a telerehabilitation study based on the Self-Monitoring, Coaching, e-Health, Interactive Feedback, and Personalization model that the intervention group exhibited significantly superior exercise adherence and duration compared to the control group. Varnfield et al [48] confirmed that smartphone-based home rehabilitation effectively enhances physical activity levels in patients with postmyocardial infarction. Optimization of daily lifestyle habits has also garnered significant attention, encompassing indicators such as medication adherence, dietary compliance, fruit and vegetable intake, sedentary time, and smoking cessation behavior. Interventions incorporating digital tools have shown positive effects on dietary adherence, sedentary time, and smoking cessation [50,51]. DHTs effectively enhance patient motivation for behavioral change through personalized reminders, real-time feedback, and adaptive goal-setting, thereby promoting the sustained maintenance of healthy behaviors.

### Patient-Reported Outcomes and Cognitive Function

Patient-reported outcomes and cognitive-related indicators constitute crucial dimensions for evaluating the humanistic value of DHTs. A total of 34 such indicators were incorporated into the studies as assessment criteria, encompassing domains such as quality of life, social cognition and support, disease knowledge, and psychological state [33-36,38-42,44-51,53,54,56,58-68,70,71,73]. Quality of life emerged as the most frequently assessed outcome, with multiple studies using scales such as the Short Form 36 Health Survey, EQ-5D, and Heart Disease-Specific Quality of Life Questionnaire. Dodson et al [53] demonstrated positive trends in mobile health interventions improving health status among older patients, while Hisam et al [54] found that mobile health interventions significantly enhanced quality of life in patients with postacute coronary syndrome. Regarding knowledge and self-management capabilities, health education emerged as the most prevalent intervention objective. Its efficacy was evaluated through indicators such as cardiovascular risk factor knowledge and self-management competence. Chan et al [34] confirmed that 0-time exercise interventions can enhance patients’ exercise self-efficacy. In the domain of social cognition and support, Duan et al [35] incorporated social cognitive outcomes into its evaluation. DHTs effectively enhance patient cognition through personalized education and interactive feedback, providing crucial support for the long-term maintenance of rehabilitation outcomes.

### Program Participation and Adherence

Participation rates and adherence in CR are core indicators for assessing the real-world feasibility of DHTs. These encompass rehabilitation program participation rates, adherence rates, completion rates, alongside patient satisfaction and perceived acceptability. Varnfield et al [48] found that smartphone-based home rehabilitation significantly improved rehabilitation uptake, adherence, and completion rates among patients with postmyocardial infarction compared to conventional rehabilitation, providing robust evidence for digital technologies enhancing rehabilitation engagement. Ramachandran et al [72] further validated the advantages of home-based remote rehabilitation in improving rehabilitation use rates. Patient satisfaction, acceptability, and perceived ease of use of the technology are also crucial evaluation components. Studies by Bernal-Jiménez et al [51] and Cruz-Cobo et al [50] both incorporated application satisfaction into their evaluation frameworks. Multiple studies indicate that DHTs, through their accessibility, convenience, and interactive features, significantly reduce participation barriers such as geographical constraints and time conflicts, laying a solid foundation for improving rehabilitation participation rates and adherence.

### Technical Feasibility, Safety, and Use

Against the backdrop of rapid advancements in DHTs, evaluating their feasibility, safety, and impact on health care service use is particularly crucial. Assessments of technical feasibility encompass device operational stability, data collection integrity, and user-friendliness. Wohlfahrt et al [71] demonstrated in their study that smart device step tracking exhibits good feasibility and compliance among patients with postmyocardial infarction. Safety assessments involve adverse event monitoring, data privacy protection, and risk alert mechanisms. Ma et al [40] demonstrated in a long-term follow-up study that digital interventions did not increase the risk of major adverse cardiovascular events. Health care use metrics include readmission rates and emergency department visit rates. Widmer et al [55] observed a downward trend in readmissions and emergency visits within the digital intervention group, though this did not reach statistical significance. Several studies have mentioned the need for cost-effectiveness analysis of digital rehabilitation, and preliminary explorations suggest that it may have potential economic advantages, but more empirical evidence is needed, though further evidence accumulation remains necessary.

## Discussion

### Principal Findings

In this study, we used a scoping review methodology to systematically evaluate the current application of DHTs in CR for patients with CHD. The research revealed its core characteristics, including diverse forms of technological application, multidimensional intervention objectives, and multilevel assessment indicators. It integrated 3 categories of technological application forms, 4 categories of intervention objectives, and 5 types of outcome assessment indicators. Findings indicate that DHTs have evolved from supplementary aids to systematic solutions, effectively overcoming the temporal and spatial constraints of traditional rehabilitation. This advance has significantly improved patient engagement in CR and treatment adherence in patients with CHD.

### Diversity and Integration of DHTs

Through this scoping review, we found that DHTs show obvious diversity and integration in the form of technology app, which can be divided into 3 main types: digital health tools, real-time remote support, and asynchronous communication. These encompass 10 specific formats including wearable devices, app, website, digital video, social media platform, telephone, group meeting, remote counseling sessions, email, and SMS text messaging. In this scoping review, we found that apps are the main intervention tool for digital CR, and wearable devices are key for real-time data monitoring; their combined application represents the most prevalent model.

From a technical support perspective, digital health tools are primarily oriented toward patient self-management and health monitoring. This aligns with the findings of van Olmen et al [75], who concluded that digital health tools can effectively empower individuals to engage in self-management and advance the achievement of relevant health goals. Real-time remote support emphasizes direct interpersonal interaction between clinicians and patients, preserving the inherent humanistic care inherent in traditional health care [76]. For instance, studies by Ryan et al [77] integrated empathy and care into remote interactions, revealing no significant difference in perceived humanistic care compared to in-person consultations. Asynchronous communication, leveraging flexible information delivery, provides patients with continuous health reminders and educational support [15].

These 3 complementary levels synergistically construct a multitiered, multidimensional, and comprehensive rehabilitation support system spanning patient self-management to real-time clinician-patient interaction. This provides a viable pathway for developing personalized, multimodal CR models. Notably, some studies further integrate offline face-to-face guidance, forming a blended rehabilitation model combining online and offline approaches [37,38,54,56]. This aligns with findings from Thomas et al [78], confirming that a comprehensive digital technology app significantly enhances the individualized adaptability of CR. Compared to traditional rehabilitation methods, this blended model partially addresses limitations such as relatively monotonous formats and insufficient consideration of individual differences [79].

### The Multifaceted Application Objectives of DHTs

In this scoping review, we found that DHTs exhibit multifaceted features in terms of intervention goals, which can be divided into 4 main dimensions: motivation and guidance, foundation of knowledge and skills, monitoring and security, and social and group dynamics. A total of 15 specific objectives have been identified. Health education, data monitoring, and reminders and alerts form the core layer; feedback, goal setting, and personalized interventions constitute the secondary layer; while some studies incorporate distinctive features such as gamification, rewards, and social support.

All interventions integrate multidimensional objectives, with health education appearing most frequently, highlighting the central role of knowledge transfer [80]. Features like gamification and social support effectively address patient issues such as lack of motivation and difficulty sustaining behavior [41,50,57]. This aligns with the WHO Global Digital Health Strategy’s advocacy for “patient-centered approaches to achieve sustainable behavioral change” [17]. This multidimensional goal integration enables DHTs to systematically tackle key barriers to participation in traditional CR [78]. Knowledge gaps can be addressed through health education, motivation deficits remedied by gamified incentives, and sustained support ensured via social interaction [81]. It is precisely this composite application model that has evolved DHTs from fragmented tools into systematic rehabilitation support systems. This significantly enhances the comprehensiveness and continuity of rehabilitation interventions, providing a crucial pathway for advancing patients with CHD understanding of CR and enabling precision-targeted interventions [15].

### Effect Evaluation

Analysis of multidimensional assessment indicators across 43 included studies demonstrates that DHTs exhibit clear short-term intervention value for CR of patients with CHD. However, evidence for long-term clinical outcomes remains scarce, strongly aligning with the findings of positive short- to medium-term effects and insufficient long-term evidence.

Regarding clinical physiological indicators, DHTs significantly improve patients’ cardiopulmonary function and physical fitness levels, with statistically significant improvements in core metrics like peak oxygen uptake and 6-minute walk distance observed in intervention groups. They also positively influence blood pressure and lipid control, validating the effectiveness of real-time monitoring and personalized guidance in short-term physiological optimization [39,42,44,49,58,66,70,71]. At the health behavior level, digital technologies significantly enhance rehabilitation adherence in areas like exercise, medication, and diet through mechanisms such as scheduled reminders and real-time feedback. They also correct unhealthy lifestyle habits like prolonged sitting, aligning with findings where health behaviors serve as core assessment dimensions [41,48,51]. Regarding patient-reported outcomes and rehabilitation service use, digital interventions effectively improve patients’ quality of life, disease awareness, and self-efficacy. They also significantly overcome temporal and spatial constraints, increasing rehabilitation participation and completion rates while alleviating barriers to traditional rehabilitation, consistent with outcome-related findings [48,53,54,72].

However, existing studies have not reached a unified conclusion regarding the assessment of long-term clinical outcomes. While some studies observed a downward trend in readmission rates, they failed to demonstrate statistical significance in reducing major adverse cardiovascular events or long-term mortality [40,47,55]. This is closely related to the limited sample sizes and short follow-up periods in most studies, as well as the significant heterogeneity in intervention designs and the lack of systematic long-term rehabilitation management systems. Research on the long-term cost-effectiveness and sustainability of these interventions is also scarce, necessitating further exploration [82].

### Advantages and Challenges of DHT

The most significant advantage of DHTs lies in their ability to effectively overcome geographical constraints and economic barriers, substantially enhancing the accessibility of CR and patient participation rates [21]. In this study, we found that by providing easily accessible, user-friendly, and highly interactive digital technologies, it is possible to significantly reduce structural barriers commonly encountered in traditional rehabilitation models, such as transport difficulties, time conflicts, and uneven resource distribution [4]. Research by Varnfield et al [48] confirmed that smartphone-based home rehabilitation significantly outperformed traditional rehabilitation in terms of uptake, adherence, and completion rates among patients with postmyocardial infarction. Ramachandran et al [72] further validated the superiority of home-based remote rehabilitation in enhancing rehabilitation use rates. This finding provides a pathway to high-quality rehabilitation services for remote areas with scarce medical resources and for patients with CHD with limited mobility, positioning DHTs as a crucial strategic tool for bridging geographical disparities in health care resources and advancing equity in cardiovascular health services. However, the widespread adoption of digital technologies also presents a new challenge: the “digital divide” [15]. Older adults, low-income groups, and patients with lower educational attainment may encounter significant difficulties in operating digital devices, using apps, or accessing information [17,83,84].

Therefore, advancing DHT must prioritize equity and inclusivity as core principles [17]. Simplified interfaces and voice-assisted features tailored for older people and low-skilled users should be developed, alongside personalized, face-to-face training in digital technology use [85]. Exploring device subsidies or digital equipment loan schemes for vulnerable groups is essential to overcome digital barriers and encourage active participation in digital CR [83]. Furthermore, key challenges for scaling DHTs include technical feasibility, data security and privacy protection, sustainable cost-effectiveness, and seamless integration of digital interventions into existing clinical workflows [15]. Currently, while some studies have begun examining implementation-level indicators for DHTs, such as the feasibility of smart device monitoring and remote rehabilitation, overall evidence remains limited. Greater practical research and systematic evaluation are urgently needed to advance the standardized application and long-term development of digital CR models [71,86].

### Limitations

Although we systematically reviewed the current application of DHTs in CR for patients with CHD through a scoping review methodology, several limitations remain. First, regarding literature sources, we only included peer-reviewed empirical research published in English. While this approach helps ensure the quality of included studies, it may overlook important literature published in other languages and relevant gray literature, thereby affecting the comprehensiveness of the study conclusions. Second, significant methodological heterogeneity among the included studies limited the integration and comparability of results. We varied significantly in intervention design, technology combinations, intervention duration, use frequency, participant characteristics, selected outcome measures, and follow-up periods. Furthermore, most studies featured small sample sizes and short follow-up durations, lacking comprehensive assessments of long-term clinical outcomes, cost-effectiveness, intervention sustainability, and impacts on health equity. This limits a thorough evaluation of the long-term value of DHTs.

### Conclusions

In this study, we used a scoping review methodology to systematically examine the current application and practical value of DHTs in CR for patients with CHD. Findings confirm that DHTs effectively improve patients’ short-term physiological function and optimize health behaviors. Simultaneously, they overcome limitations in traditional CR regarding spatial-temporal constraints and health care resource allocation, significantly enhancing patient engagement and adherence to rehabilitation programs. In clinical practice, health care providers can integrate multiple DHTs to develop personalized rehabilitation plans tailored to individual characteristics such as patient age, digital literacy, and disease severity, thereby enhancing the precision and adaptability of CR. Future research should prioritize large-scale, multicenter, long-term follow-up randomized controlled trials to thoroughly investigate the impact of DHTs on long-term clinical outcomes for patients with CHD and explore potential mechanisms of action, such as long-term mortality and major adverse cardiovascular events. This will provide more robust evidence-based support for validating their long-term efficacy and advancing standardized clinical implementation.

## Supplementary material

10.2196/85917 Multimedia Appendix 1 Search strategies for each database.

10.2196/85917 Checklist 1 PRISMA-ScR checklist.

## Abbreviations

CHD: coronary heart disease
CR: cardiac rehabilitation
CVD: cardiovascular disease
DHT: digital health technology
PRISMA: Preferred Reporting Items for Systematic Reviews and Meta-Analyses
PRISMA-S: extension to the PRISMA Statement for Reporting Literature Searches in Systematic Reviews
PRISMA-ScR: Preferred Reporting Items for Systematic Reviews and Meta-Analyses extension for Scoping Reviews
WHO: World Health Organization
