# Supplementary material for: Application of Digital Health Technologies in Cardiac Rehabilitation for Patients With Coronary Heart Disease: Scoping Review
Source: J Med Internet Res. 2026 Apr 29;28:e85917. doi: 10.2196/85917 (PMC13128058; doi:10.2196/85917)
Supplement: Multimedia Appendix 1 [file jmir-v28-e85917-s001.pdf]

Pubmed Search strategy：

((((((((((((((("Coronary Disease"[Mesh]) OR ("Myocardial Infarction"[Mesh])) OR ("Coronary Artery Disease"[Mesh])) OR ("Coronary Heart Disease*"[Title/Abstract])) OR ("Heart Attack*"[Title/Abstract])) OR ("Myocardial Infarct*"[Title/Abstract])) OR ("Cardiovascular Stroke*"[Title/Abstract])) OR ("acute coronary syndrome"[Title/Abstract])) OR ("angina pectoris"[Title/Abstract])) OR ("STEMI"[Title/Abstract])) OR ("NSTEMI"[Title/Abstract])) OR ("PCI"[Title/Abstract])) OR ("percutaneous coronary intervention"[Title/Abstract])) OR ("CABG"[Title/Abstract])) OR ("coronary artery bypass grafting"[Title/Abstract]))

AND

((((((((((((((((((((((((((((((("Telemedicine"[Mesh]) OR ("Wearable Electronic Devices"[Mesh])) OR ("Digital Health"[Mesh])) OR ("Remote Patient Monitoring"[Mesh])) OR ("Text Messaging"[Mesh])) OR ("Virtual Medicine"[Title/Abstract])) OR ("Tele-Referral*"[Title/Abstract])) OR ("Mobile Health"[Title/Abstract])) OR ("mHealth"[Title/Abstract])) OR ("Telehealth"[Title/Abstract])) OR ("eHealth"[Title/Abstract])) OR ("Tele Intensive Care"[Title/Abstract])) OR ("Tele Care"[Title/Abstract])) OR ("Wearable Device*"[Title/Abstract])) OR ("Wearable Technolog*"[Title/Abstract])) OR ("Wearable Computer"[Title/Abstract])) OR ("Digital Health Technolog*"[Title/Abstract])) OR ("Short Message Service"[Title/Abstract])) OR ("Health Technolog*"[Title/Abstract])) OR ("Short Message Service"[Title/Abstract])) OR ("digital therapeutics"[Title/Abstract])) OR ("smartwatch"[Title/Abstract])) OR ("fitness tracker"[Title/Abstract])) OR ("activity tracker"[Title/Abstract])) OR ("tele-rehabilitation"[Title/Abstract])) OR ("virtual care"[Title/Abstract])) OR ("mobile phone"[Title/Abstract])) OR ("cell phone"[Title/Abstract])) OR ("application"[Title/Abstract])) OR ("internet-based"[Title/Abstract])) OR ("web-based"[Title/Abstract])) OR ("online program"[Title/Abstract])))

AND

((((((((((((((((("Cardiac Rehabilitation"[Mesh]) OR ("Secondary Prevention"[Mesh])) OR ("Cardiac Rehabilitation*"[Title/Abstract])) OR ("Cardiovascular Rehabilitation*"[Title/Abstract])) OR ("Secondary Prevention*"[Title/Abstract])) OR ("Disease Prevention*"[Title/Abstract])) OR ("Secondary Disease Prevention*"[Title/Abstract])) OR ("Early Therap*"[Title/Abstract])) OR ("Relapse Prevention*"[Title/Abstract])) OR ("exercise training"[Title/Abstract])) OR ("physical activity"[Title/Abstract])) OR ("lifestyle modification"[Title/Abstract])) OR ("behavior change"[Title/Abstract])) OR ("self-management"[Title/Abstract])) OR ("Exercise Therapy"[Title/Abstract])) OR ("Patient Education"[Title/Abstract])) OR ("Risk Factor Management"[Title/Abstract])) OR ("Medication Adherence"[Title/Abstract]))

Embase Search strategy：

('coronary disease':ti,ab,kw OR 'myocardial infarction':ti,ab,kw OR 'coronary artery disease':ti,ab,kw OR 'coronary heart disease*':ti,ab,kw OR 'heart attack*':ti,ab,kw OR 'myocardial infarct*':ti,ab,kw OR 'cardiovascular stroke*':ti,ab,kw OR 'acute coronary syndrome':ti,ab,kw OR 'angina pectoris':ti,ab,kw OR 'stemi':ti,ab,kw OR 'nstemi':ti,ab,kw OR 'pci':ti,ab,kw OR 'percutaneous coronary intervention':ti,ab,kw OR 'cabg':ti,ab,kw OR 'coronary artery bypass grafting':ti,ab,kw)

AND

('telemedicine':ti,ab,kw OR 'wearable electronic devices':ti,ab,kw OR 'digital health':ti,ab,kw OR 'remote patient monitoring':ti,ab,kw OR 'text messaging':ti,ab,kw OR 'virtual medicine':ti,ab,kw OR 'tele-referral*':ti,ab,kw OR 'mobile health':ti,ab,kw OR 'mhealth':ti,ab,kw OR 'telehealth':ti,ab,kw OR 'ehealth':ti,ab,kw OR 'tele intensive care':ti,ab,kw OR 'tele care':ti,ab,kw OR 'wearable device*':ti,ab,kw OR 'wearable technolog*':ti,ab,kw OR 'wearable computer':ti,ab,kw OR 'digital health technolog*':ti,ab,kw OR 'health technolog*':ti,ab,kw OR 'short message service':ti,ab,kw OR 'digital therapeutics':ti,ab,kw OR 'smartwatch':ti,ab,kw OR 'fitness tracker':ti,ab,kw OR 'activity tracker':ti,ab,kw OR 'tele-rehabilitation':ti,ab,kw OR 'virtual care':ti,ab,kw OR 'mobile phone':ti,ab,kw OR 'cell phone':ti,ab,kw OR 'application':ti,ab,kw OR 'internet-based':ti,ab,kw OR 'web-based':ti,ab,kw OR 'online program':ti,ab,kw)

AND

('cardiac rehabilitation':ti,ab,kw OR 'secondary prevention':ti,ab,kw OR 'cardiac rehabilitation*':ti,ab,kw OR 'cardiovascular rehabilitation*':ti,ab,kw OR 'secondary prevention*':ti,ab,kw OR 'disease prevention*':ti,ab,kw OR 'secondary disease prevention*':ti,ab,kw OR 'early therap*':ti,ab,kw OR 'relapse prevention*':ti,ab,kw OR 'exercise training':ti,ab,kw OR 'physical activity':ti,ab,kw OR 'lifestyle modification':ti,ab,kw OR 'behavior change':ti,ab,kw OR 'self-management':ti,ab,kw OR 'exercise therapy':ti,ab,kw OR 'patient education':ti,ab,kw OR 'risk factor management':ti,ab,kw OR 'medication adherence':ti,ab,kw)

**Cochrane Library Search strategy：**	
#1	MeSH descriptor: [Coronary Disease] explode all trees
#2	MeSH descriptor: [Myocardial Infarction] explode all trees
#3	MeSH descriptor: [Coronary Artery Disease] explode all trees
#4	Coronary Heart disease* OR Heat Attack* OR Mvocardial infarct* 0R Cardiovascular Stroke* OR acute coronary syndrome OR "angina pectoris" OR"STEMI" OR "'NSTEMI" OR "PCI" OR "percutaneous coronary intervention" OR "CABG" OR "coronary artery bypass grafting"
#5	#1 OR #2 OR #3 OR #4
#6	MeSH descriptor: [Telemedicine] explode all trees
#7	MeSH descriptor: [Wearable Electronic Devices] explode all trees
#8	MeSH descriptor: [Digital Health] explode all trees
#9	MeSH descriptor: [Remote Patient Monitoring] explode all trees
#10	MeSH descriptor: [Text Messaging] explode all trees
#11	"Virtual Medicine" OR Tele-Referral* OR "Mobile Health" OR "mHealth" OR "Telehealth" OR "eHealth" OR "Tele Intensive Care" OR "Tele Care" OR Wearable Device* OR Wearable Technolog* OR "Wearable Computer" OR Digital Health Technolog* OR Health Technolog* OR "Short Message Service" OR "digital therapeutics" OR "smartwatch" OR "fitness tracker" OR "activity tracker" OR "tele-rehabilitation" OR "virtual care" OR "mobile phone" OR "cell phone" OR "application" OR "internet-based" OR "web-based" OR "online program"
#12	#6 OR #7 OR #8 OR #9 OR #10 OR #11
#13	MeSH descriptor: [Cardiac Rehabilitation] explode all trees
#14	MeSH descriptor: [Secondary Prevention] explode all trees
#15	Cardiac Rehabilitation* OR Cardiovascular Rehabilitation* OR Secondary Prevention* OR Disease Prevention* OR Secondary Disease Prevention* OR "Early Therap*" OR Relapse Prevention* OR "exercise training" OR "physical activity" OR "lifestyle modification" OR "behavior change" OR "self-management" OR "Exercise Therapy" OR "Patient Education" OR "Risk Factor Management" OR "Medication Adherence"
#16	#13 OR #14 OR #15
#17	#5 AND #12 AND #16

Web of Science & EBSCO Search strategy：

("Coronary Disease" OR "Myocardial Infarction" OR "Coronary Artery Disease" OR "Coronary Heart Disease*" OR "Heart Attack*" OR "Myocardial Infarct*" OR "Cardiovascular Stroke*" OR "acute coronary syndrome" OR "angina pectoris" OR "STEMI" OR "NSTEMI" OR "PCI" OR "percutaneous coronary intervention" OR "CABG" OR "coronary artery bypass grafting").ab,kw,ti.
AND
("Telemedicine" OR "Wearable Electronic Devices" OR "Digital Health" OR "Remote Patient Monitoring" OR "Text Messaging" OR "Virtual Medicine" OR "Tele-Referral*" OR "Mobile Health" OR "mHealth" OR "Telehealth" OR "eHealth" OR "Tele Intensive Care" OR "Tele Care" OR "Wearable Device*" OR "Wearable Technolog*" OR "Wearable Computer" OR "Digital Health Technolog*" OR "Health Technolog*" OR "Short Message Service" OR "digital therapeutics" OR "smartwatch" OR "fitness tracker" OR "activity tracker" OR "tele-rehabilitation" OR "virtual care" OR "mobile phone" OR "cell phone" OR "application" OR "internet-based" OR "web-based" OR "online program").ab,kw,ti.
AND
("Cardiac Rehabilitation" OR "Secondary Prevention" OR "Cardiac Rehabilitation*" OR "Cardiovascular Rehabilitation*" OR "Secondary Prevention*" OR "Disease Prevention*" OR "Secondary Disease Prevention*" OR "Early Therap*" OR "Relapse Prevention*" OR "exercise training" OR "physical activity" OR "lifestyle modification" OR "behavior change" OR "self-management" OR "Exercise Therapy" OR "Patient Education" OR "Risk Factor Management" OR "Medication Adherence").ab,kw,ti.
